# Diffuse alveolar damage, acute respiratory distress syndrome (ARDS), and non-cardiogenic pulmonary edema (NCPE). Part 2: NCPE apart from ARDS, molecular and cellular consequences of primary injury to the lung interstitium, with comparative immunology of the dog and cat

**DOI:** 10.1177/10406387251415457

**Published:** 2026-03-12

**Authors:** Hannah E. Wong, Joseph P. Boyle

**Affiliations:** Department of Veterinary Medicine, University of Cambridge, Cambridge, UK

**Keywords:** cell death, endotype, histology, inflammasome, innate immunity, lung damage

## Abstract

Diffuse alveolar damage (DAD) and the related clinical syndrome—acute respiratory distress syndrome (ARDS)—result in non-cardiogenic pulmonary edema (NCPE) through acute endothelial and/or alveolar epithelial injury. ARDS endotypes that have been suggested for human medicine may be applicable to veterinary contexts, and the molecular signatures of those endotypes require classification. Both the primary injury and the subsequent amplification by secondary inflammatory processes contribute to the molecular signature; raising awareness of these primary and secondary input processes increases the resolution of data analysis. Furthermore, species-specific differences in inflammatory pathways should be considered when interpreting data or applying new precision immunomodulatory therapeutics. We review other causes of NCPE in dogs and cats and explore the challenges in diagnosing pathologic pulmonary edema with histology. We explore the subsequent immune and cell-death processes that amplify the primary insult to the lung interstitium, ultimately leading to ARDS or DAD, along with the comparative immunology. For cases of acute lung damage—which result in non-cardiogenic edema that lack the classical histologic features of DAD—the histologic changes in dogs and cats can be subtle and nonspecific. Combining these subtle findings with a multidisciplinary approach to reviewing case evidence can yield greater diagnostic accuracy. Therefore, we summarize the histologic features that support a diagnosis of pathologic pulmonary edema and differentiate this condition from peracute agonal pulmonary edema or artefact.

The acute respiratory distress syndrome (**ARDS**) endotypes, originally defined in the context of human medicine, separate ARDS etiologies into 5 groups. Those 5 groups, which are classified by the primary injury, are *epithelial*, *endothelial*, *local inflammatory response*, *angiopathy*, and *systemic inflammatory response*. The premise of the ARDS endotypes is that the link between the development of ARDS and the original primary injury is context-specific and, therefore, is differentiable on molecular data. Currently, ARDS endotypes are conceptual in both human and veterinary medicine. The molecular signatures of the ARDS endotypes are yet to be determined.^10^ Understanding the inflammatory pathways that amplify the primary injury is important to maximize accurate interpretation of research data. In this review and Part 1,^98^ we present processes believed to contribute to the pathogenesis of ARDS in veterinary medicine, based on the current literature. Our aim is to support experimental design by advancing ARDS endotypes from concepts to defined entities, recognizing that the parameters of those entities may ultimately differ from the content that we present. Furthermore, compared with humans, dogs and cats lack certain components of the inflammatory and cell death pathways, which impacts the interpretation of research data and the application of precision therapeutics in these species.

ARDS is a type of non-cardiogenic pulmonary edema (**NCPE**) that involves alveolar-endothelial barrier dysfunction. Other cases of NCPE may develop ARDS as a secondary acute deterioration. Acute lung damage (**ALD**) that induces NCPE—but lacks the histologic features of diffuse alveolar damage (**DAD**) in dogs and cats—can be subtle and nonspecific. In those cases, to ensure that histologic evidence of ALD is appreciated at histologic review, a multidisciplinary approach can offer additional corroborating evidence for reaching a shared diagnosis.

After reading this review, readers should be able to:

- Describe the pathophysiology of NCPE.
- Summarize histologic evidence to help differentiate pathologic pulmonary edema from peracute agonal pulmonary edema or artefact.
- Describe the cellular and molecular consequences of primary injury to the lung interstitium that support ARDS development.
- Summarize the comparative immunology of select pattern recognition receptor (**PRR**) pathways that have roles in ARDS development in humans, mice, cats, and dogs.

## Non-cardiogenic pulmonary edema apart from ARDS and DAD

ARDS is a type of NCPE accompanied by acute-onset severe hypoxemia and is defined by specific clinical and biochemical thresholds; DAD is histologic evidence of a type of NCPE. In addition to ARDS and DAD, NCPE has many other causes, and these etiologies can be grouped by pathogenesis: increased hydrostatic pressure, increased permeability of alveolar barrier, disruption of active fluid transport out of alveoli, reduced oncotic pressure, and increased interstitial hydrostatic pressure. In the following section, we review the common causes of NCPE, as grouped by pathogeneses listed above. We then discuss the typical macroscopic and microscopic appearance of NCPE.

### Causes of NCPE

Common causes of increased permeability of the alveolar barrier include sepsis or endotoxemia, anaphylaxis, histamine release induced by drugs or from mast cell tumors, and mechanical ventilation.^17^ Increased permeability of the alveolar barrier and disruption of active fluid transport out of alveoli mirror the mechanism of injury in endothelial and epithelial ARDS endotypes, respectively.^98^

Causes of pulmonary hypertension resulting in pulmonary edema are summarized in the American College of Veterinary Internal Medicine (ACVIM) classification of canine pulmonary hypertension (**Table 1**).^69^ Further subsets of non-cardiogenic pulmonary edema are negative-pressure pulmonary edema (**NPPE**) and iatrogenic volume overload. The pathogenesis of NPPE is multifactorial and includes both pulmonary pressure changes and epithelial damage.^43^ The high-negative intrathoracic pressure generated to overcome upper airway obstruction results in increased pulmonary hydrostatic pressure caused by increased venous return to the right side of the heart and decreased alveolar pressure. These factors create a large pressure gradient that moves liquid from lung capillaries into airspaces. The mechanical stress on alveoli also contributes to epithelial damage and epithelial-endothelial barrier dysfunction.^43^ In a review of 35 clinical NPPE cases in dogs, causes included lead pulling; near hanging; accidental choking; physical obstruction of airflow by lesions, such as neoplasms; and non-accidental airway obstruction, such as strangulation.^34^ Other causes of NCPE in dogs and cats include neurologic disease, electrocution, post-collapsed lung re-expansion, and after drowning.^86^ Radiographic findings suggestive of NCPE are interstitial-to-alveolar lung opacities in the absence of signs of left-sided congestive heart failure or fluid overload.^86^

**Table 1.** Selected causes of non-cardiogenic pulmonary edema in dogs and cats.

NCPE classification	Examples
Increased permeability of alveolar barrier	Local: NPPE
	Neurologic pulmonary edema
	Electrocution
	Systemic: sepsis, uremia (uremic pneumopathy)
Disruption of alveolar fluid active transport	Ventilator injury
	NPPE
Non-cardiogenic pulmonary arterial hypertension	Idiopathic (IPAH) or heritable
	Drugs and toxins induced
	Pulmonary vasculitis
	Pulmonary vascular amyloid deposition
	Pulmonary veno-occlusive disease (PVOD) or pulmonary capillary hemangiomatosis (PCH)
Respiratory disease, hypoxia, or both	Chronic obstructive airway disorders
	Interstitial lung disease
	Pneumonia (including organizing)
	Diffuse pulmonary neoplasia
	Sleep disordered breathing
	Chronic exposure to high altitudes
	Developmental lung disease
	Bronchiolar disorders
Pulmonary thromboemboli	Acute or chronic pulmonary emboli/thrombi/thromboemboli (PE/PT/PTE)
Parasitic disease	*Dirofilaria immitis*
	*Angiostrongylus vasorum*

NCPE = non-cardiogenic pulmonary edema, incorporating ACVIM pulmonary hypertension consensus classifications^69^; NPPE = negative pressure pulmonary edema.

### Macroscopic appearance of NCPE

The macroscopic appearance of pulmonary edema in dogs and cats consists of heavy, wet lungs that exude abundant translucent red fluid; similar fluid may pool within large airways and the trachea. Pulmonary congestion and hemorrhage can result in similar morphologic features, with the 3 processes often occurring together as a common nonspecific peracute agonal change. For most causes of NCPE, additional macroscopic or microscopic evidence is required to determine if the observed changes occurred before death. Fumonisin toxicity in pigs, African horse sickness and Hendra viruses in horses, and bluetongue virus in sheep are specific conditions in which pulmonary edema is so prominent that the macroscopic appearance is suggestive of the etiology.^17^

### Microscopic appearance of NCPE

**Figure 1.**
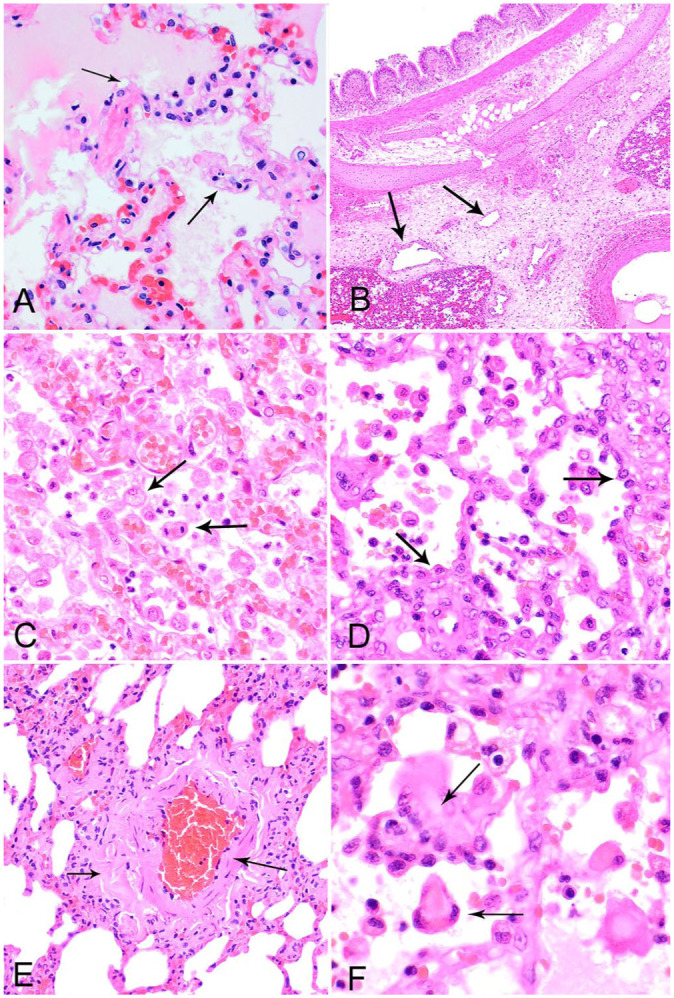
Histologic features that support a diagnosis of pathologic pulmonary edema. H&E. **A.** Alveolar septal edema, clear or wispy material expanding septa (arrows), in a dog with pulmonary thrombosis. **B.** Dilated lymphatics (arrows) in the lung interstitium in a dog with diffuse alveolar damage and myocarditis caused by parvovirus. **C.** Foamy and dividing alveolar macrophages (arrows) in a dog with pulmonary hypertension caused by a congenital heart defect. **D.** Type II pneumocyte hyperplasia (arrows) in a dog with an inhaled foreign body. **E.** Vascular remodeling, muscularization of a vein (long arrow), and perivascular fibrosis (short arrow) in a dog with pulmonary thromboembolism. **F.** Pleomorphic alveolar macrophages or type II pneumocytes may be indistinguishable (arrows) in a dog with an inhaled foreign body.

The typical microscopic appearance of pulmonary edema consists of homogeneous-to-wispy, translucent, eosinophilic material within alveolar spaces. Low-protein exudates can be nearly transparent on H&E-stained sections, and washout during tissue processing further reduces the material present on the slide. To make a diagnosis, corroborating evidence is required, which includes expanded alveolar spaces that may (or may not) contain visible content, alveolar septa and/or interstitial perivascular areas expanded by clear space or eosinophilic material (perivascular clearing; **Fig. 1A**), dilated interstitial lymphatics (**Fig. 1B**), and an obvious underlying lesion(s). Eosinophilic material in alveoli alone is not specific for pulmonary edema given that pink material is often present in alveoli of autolyzed carcasses and animals euthanized by barbituates.^17^ Alveolar macrophage number and morphology are useful visible indicators because they become large and foamy from clearing alveolar fluid and, with chronicity of the edema, proliferate (**Fig. 1C**). Other histologic features that may be found in chronic NCPE include type II pneumocyte hyperplasia (**Fig. 1D**) and interstitial fibrosis, although these features are not specific to NCPE and can be induced by many causes of interstitial damage. In cases of NCPE with pulmonary hypertension, lung hemorrhage may occur alongside pulmonary edema; hence, hemosiderophages within the alveolar space and/or lung interstitium can be corroborative evidence of pre-existing pathologic pulmonary edema. If the NCPE is related to increased hydrostatic pressure, changes to vascular walls, such as venous muscularization, arterial wall hypertrophy, and perivascular fibrosis, may be present (**Fig. 1E**).

## Diagnostic challenges encountered with NCPE and DAD

**Figure 2.**
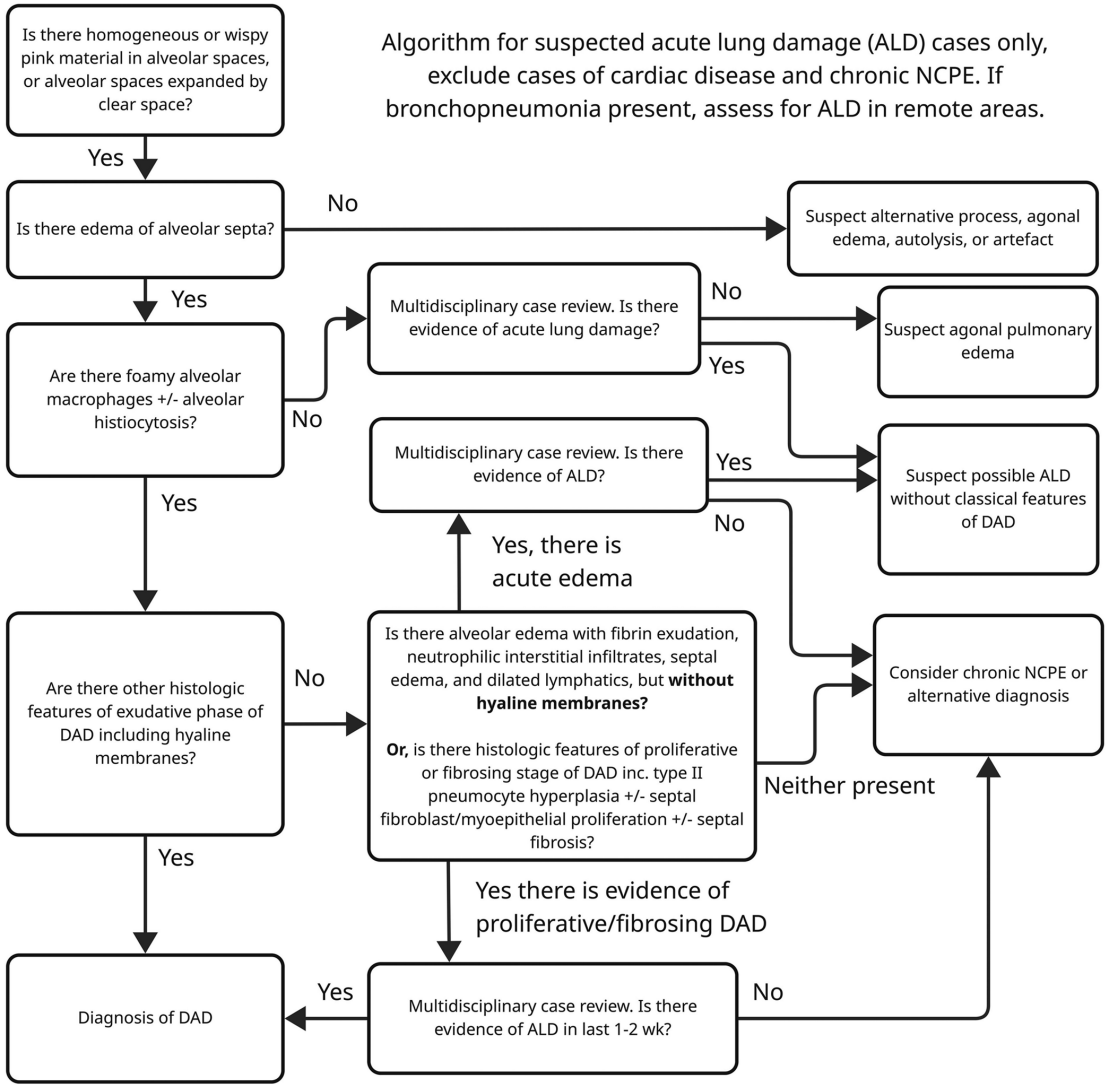
Schematic of a non-exhaustive diagnostic algorithm to support early career pathologists in the differentiation of acute lung damage (ALD) causing non-cardiogenic pulmonary edema (NCPE), including diffuse alveolar damage (DAD) from artefact and agonal pulmonary edema.

NCPE and DAD can present diagnostic challenges to pathologists. A common challenge is differentiating pathologic NCPE from artefactual and agonal pulmonary edema, and postmortem autolysis-related fluid accumulation. In contrast to the name, DAD can have a patchy distribution^74^; therefore, sampling characteristics contribute to a diagnostician’s ability to detect this condition. NCPE and DAD increase numbers of alveolar macrophages, and the macrophages have foamy cytoplasm through phagocytosis of the excess liquid (Fig. 1C). Both alveolar macrophages and type II pneumocytes can become pleomorphic in response to injury and inflammation. If hypertrophied type II pneumocytes are present in isolation or only have a tenuous hold of the alveolar basement membrane, cytokeratin and/or histiocytic IHC markers may be required to distinguish between them (**Fig. 1F**).^17^ Hyaline membranes are required for a diagnosis of DAD.^16^ Thus, a further challenge in peracute lung damage is that the histologic changes may consist only of alveolar edema and hemorrhage.^16^ A multidisciplinary approach to gather contextual information—including clinical history, physical examination, and imaging results—is valuable for interpreting the often nonspecific histologic changes in cases of suspected ALD (**Fig. 2**). Increased awareness of the hypoinflammatory ARDS subphenotype and the ARDS endotypes^11^ may support the determination of subtle histologic features of hypoinflammatory acute lung lesions in cases of the hypoinflammatory ARDS subphenotype.

A contrasting diagnostic challenge is detecting secondary DAD induced by a bronchopneumonia with extensive tissue destruction. Assessing DAD in areas spatially distinct from the bronchopneumonia would increase sensitivity but is limited by the amount of tissue available in the sample. The category of “bronchointerstitial” has been removed as a pneumonia classification from the new edition of a pathology reference textbook,^17^ which may help focus attention on secondary interstitial pneumonia as a related but separate process from primary bronchopneumonia. A further challenge is that the tissue sample for histology is often taken in chronic disease, after the primary lesion has been effaced by secondary inflammation. In these cases, assignment of an endotype may rely on retrospective inference of the likely etiology.

## Consequences of initial injury to the lung interstitium

After the primary injury to the interstitium has occured,^98^ several key processes contribute to sustained alveolar and septal edema and dysregulation of gas-exchange, which supports a transition from lung injury to ARDS. These processes are macrophage activation, a cytokine storm, and immunopathology. Macrophage activation occurs early in the immune response and is considered an important step in shaping the composition and extent of the inflammatory response, including the recruitment of other cell types.^65^ The cytokine storm occurs after resident and recruited inflammatory cells ramp up cytokine production through positive feedback loops.^40^ The composition of the cytokine storm has been found to impact clinical outcomes.^41^ Immune response in the lung has the potential to transition into immunopathology relatively easily, because effective pulmonary gas exchange relies upon the maintenance of tissue homeostasis.^38^ After exploring these cellular processes in more detail below, we review the molecular mechanisms controlling cell death processes that contribute to the pathology of ARDS.

### Macrophage activation

In the lung, there are 3 main macrophage populations, tissue-resident alveolar macrophages, interstitial macrophages, and bone-marrow-derived-monocytes that are recruited into the lung tissue.^61^ Certain species have a fourth population of pulmonary intravascular macrophages (**PIMs**), which are discussed below. Tissue-resident alveolar macrophages self-proliferate to maintain appropriate levels^54^ and have a baseline anti-inflammatory phenotype.^61^ They maintain homeostasis by resolving inflammatory threats without involving other inflammatory cell populations.^56^ When the challenge exceeds the ability to maintain homeostasis, circulating monocytes are recruited to the lung and function as a strongly pro-inflammatory subset of recruited alveolar macrophages.^54^ Activated macrophages can initiate cell death pathways in adjacent epithelial or endothelial cells, and coordinate recruitment of circulating monocytes and neutrophils through cytokine release. Alveolar macrophages are central modulators of alveolar inflammation by influencing cytokine release from local cells through paracrine signaling.^61^ For example, in an in vitro lipopolysaccharide (**LPS**) model, leukocyte-chemotactic cytokine release from alveolar endothelial cells was dependent on paracrine release of TNF and IL1β from alveolar macrophages.^84^ In a mouse model of acid inhalation, depletion of alveolar macrophages reduced TNF production and decreased TNF-induced caspase-8 dependent signaling in type I pneumocytes, resulting in reduced alveolar epithelial dysfunction and pulmonary edema.^60^ The paracrine activation is bi-directional as in the early phase of lung inflammation neutrophil extracellular traps (**NETs**) support the M1 pro-inflammatory polarization of alveolar macrophages in humans and mice, facilitating the local tissue dysregulation and progression to ARDS.^80,84^ Macrophage phagocytic function influences ARDS progression. In human cases of ARDS, alveolar macrophages demonstrated reduced clearance of apoptotic cells (efferocytosis), which was associated with increased alveolar inflammation and reduced survival.^50^

PIMs add additional species-dependent modulation. PIMs were detected initially in rats,^94^ with constitutive PIMs then found in sheep, calves, pigs, goats, horses, cats, and whales, but absent in monkeys and chickens.^12,22,25^ PIMs are inducible in rodents and rabbits. PIMs have also been induced in human cases of hepato-pulmonary syndrome^73^ and in dogs with acute necrotizing pancreatitis.^91^ Constitutive PIMs are firmly attached to the capillary endothelium in alveolar septa,^96^ and have phagocytic, inflammatory, and thrombotic effects induced by blood-borne and airway challenges.^7,73^ Phagocytosis by PIMs is rapid and incorporates pathogens, erythrocytes, fibrin, cellular debris, and immune cells.^7^ The presence (or absence) of PIMs in a species may influence sensitivity to lung inflammation. The depletion of PIMs in calves challenged with *Mannheimia haemolytica* was protective against ALD,^77^ whereas PIM-ablated horses were only partially protected from LPS-induced lung inflammation.^59^ Bovine PIMs activated platelets in response to inhaled *M. haemolytica*.^77^ On review, the authors suggested that PIM-activated platelets sequester activated neutrophils in the lungs, thereby initiating lung injury.^73^

### Cytokine storm

A cytokine storm supports the transition from lung injury to ARDS or DAD.^71^ The earlier concept of a cytokine storm—composed of positive feedback of pro-inflammatory mediators—has now been replaced with the recognition that inflammation and immunosuppression occur concurrently, termed persistent inflammation, immunosuppression, and catabolism syndrome (**PICS**).^33^ The composition of the PICS cytokine storm is influenced by etiologic factors, such as virus viability, given that in vitro infection of human alveolar macrophages requires viable respiratory syncytial virus to produce TNF (an important cytokine in lung injury), whereas IL6 and IL8 are produced irrespective of virus viability.^6^

Analysis of cytokines and related biomarkers from human clinical studies has identified hyperinflammatory and hypoinflammatory ARDS subphenotypes.^14,81^ A pediatric ARDS cohort study recapitulated divisions from adult studies,^81^ separating patients into hyperinflammatory and hypoinflammatory subphenotypes.^100^ Biomarkers with the greatest differences included IL6, IL8, soluble TNF receptor 1 (sTNFR1), sTNFR2, granzyme B, angiopoietin-2, sRAGE, and matrix metalloprotease 8, which were present at higher levels in the hyperinflammatory phenotype and were in good agreement among independent cohorts.^100^

**Figure 3.**
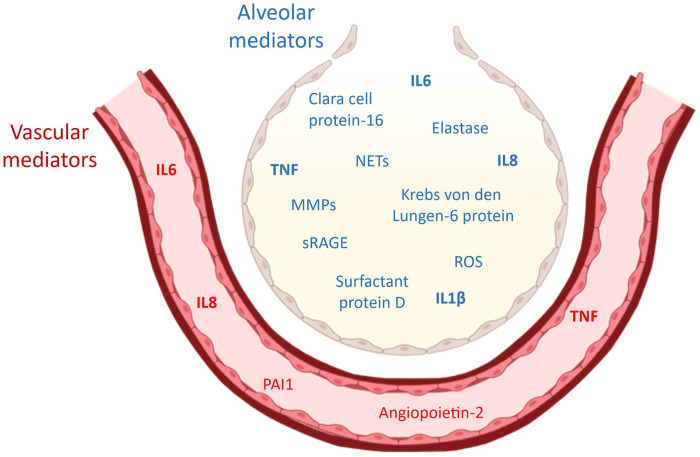
Selected ARDS biomarkers grouped by their primary compartment of release, alveolar versus vascular, based on data from human observational studies. Biomarkers in bold are differentially elevated in the hyperinflammatory ARDS subphenotype compared with the hypoinflammatory subphenotype. MMPs = matrix metalloproteinases; NETs = neutrophil extracellular traps; sRAGE = soluble receptor for advanced glycation end products; PAI1 = plasminogen activator inhibitor 1.

Cytokines can also offer evidence on the degree of compartmentalization between the alveolar and systemic vascular compartments. This has practical applications, helping to determine the molecular characteristics of the ARDS endotypes. A category, *extrapulmonary ARDS*, captures the causes of ARDS that originate outside the lungs, including those classified as *systemic inflammatory response ARDS endotype* and *angiopathy ARDS endotype*. Based on human data, key inflammatory mediators—caused primarily by alveolar inflammation verses extrapulmonary sources—have been identified in ARDS (**Fig. 3**).^11,81^ Key mediators in alveolar-based ARDS include IL1β, IL6, IL8, and TNF from alveolar macrophages; NETs, reactive oxygen species (**ROS**), matrix metalloproteinases (**MMPs**), and elastase from recruited neutrophils in alveoli; surfactant protein D and Krebs von den Lungen-6 protein from type II pneumocytes; and sRAGE and Clara cell protein 16 from type I pneumocytes. Extrapulmonary ARDS that involves systemic vascular inflammation is characterized by increased circulatory IL6, IL8, and TNF, angiopoietin-2, plasminogen activator inhibitor 1, and a reduction in protein C. When both systemic inflammation and local alveolar inflammation are present—for example, pneumonia causing sepsis that then progresses to ARDS—combinations of those cytokines are detected.

The use of lung-protective low-tidal-volume ventilation in human ICU patients has reduced the effect of inflammatory cytokines in both the airspace and plasma compartments. This suggests that either translocation of the cytokines from the pulmonary compartment to the circulation, or additional cytokines released in response to the mechanical damage is an important stage in the generation of the cytokine storm.^66^

Certain cytokines can enhance the pro-inflammatory response through wide-ranging paracrine effects on other immune cells via innate immune receptors. Two cytokines that are major effectors of paracrine cytokine release are TNF and IL1β. When inhibited for TNF and/or IL1β activity, human alveolar macrophages infected with influenza A virus significantly decreased production of the proinflammatory cytokines CCL5 and CXCL8.^92^ A poxvirus-produced TNF receptor homolog reduced proinflammatory cytokine levels and reduced lung lesions in an acute pneumonia mouse model of ectromelia infection.^2^ IL1β undergoes proteolytic activation predominantly by cytosolic caspases and, once released by secretion or lytic cell death,^83^ activates the IL1 receptor expressed on innate immune cells, such as neutrophils, monocytes, or macrophages, resulting in pro-inflammatory effects.^31^ Neutrophil and mast cell-derived proteases can also catalyze the proteolytic activation of pro-IL1β, the inactive precursor, when it is released into the extracellular space after efflux or inflammatory cell death, further contributing to a ramped-up pro-inflammatory response.^1^ This ramp up can affect clinical severity, given that *Streptococcus pneumoniae* strains, which are naturally deficient in IL1β induction because of the loss of the toxin pneumolysin,^97^ are associated with reduced mortality in human cases of pneumonia compared with strains that induce IL1β.^78^

### Immunopathology

Many processes contribute to immune-mediated lesions occurring within ARDS, and these include off-target inflammatory cell products, excessive activation of pro-inflammatory cell-signaling pathways, and activation of complement and coagulation.

Recruited neutrophils cause bystander damage to type I and type II pneumocytes by releasing ROSs, NETs, proteases, prostaglandins, and leukotrienes, leading to disruption of the alveolar-capillary interface and alveolar edema.^13^ Processes driving immunopathology can become decoupled from the initiating pathogen. For example, in a mouse model of inhaled ectromelia virus acute pneumonia, late-stage antiviral treatment reduced viral load but did not reduce lung lesions, whereas late-stage treatment with antivirals and TNF-blockers reduced lung damage and clinical disease.^58^

The transcription factor NF-κB is a major regulatory factor in lung inflammation because it is integral to the signaling pathways of many pattern recognition receptors (**PRRs**).^62,79^ Targeted knockdown of NF-κB in an in vitro model of pneumococcal pneumonia resulted in reduced cytokine release, neutrophil recruitment, and bacterial killing^64^; knock-out of MyD88, a primary signaling molecule in the NF-κB pathway, blocked macrophage TNF responses to in vitro *S. pneumoniae* infection.^99^

Activation of complement by innate inflammation and its subsequent induction of immunopathology is another factor that contributes to the development of ARDS,^3^ with complement inhibitors having a therapeutic effect in people with COVID–19-related ARDS.^32^

Immune-mediated lesions also occur through the activation of the coagulation system. In the lung, tissue factor is considered to be the primary initiator of coagulation in response to severe infection.^87^ Activated alveolar epithelial cells release microparticles containing tissue factor, facilitating local coagulation and fibrin deposition.^5^ The deposition of fibrin in alveoli contributes to severe lung injury^37^; tissue factor inhibitors reduced lung damage in a primate sepsis-induced experimental acute lung injury (**ALI**) model.^95^

## The cell death processes of ARDS and DAD

Cell death processes can be categorized as regulated or non-regulated, and then further categorized depending on the immediate downstream effect, and whether those processes are immunogenic or non-immunogenic. Known types of cell-death pathways involved in ARDS include apoptosis, necrosis, pyroptosis, ferroptosis, and necroptosis. Understanding ARDS endotypes and associated inflammatory and cell-death pathways have important clinical implications in veterinary medicine, supporting the identification of diagnostic and/or prognostic biomarkers, and personalized treatments. Although targeted therapies are not yet widely available, applications in human medicine are being developed, which likely will inspire and create new opportunities for veterinary therapeutics. For example, immunomodulatory molecules targeting inflammatory or cell-death pathways are in pre-clinical trials for human sepsis,^9,57^ and mesenchymal stromal cells are in clinical translational studies as a treatment for human ARDS.^20^

### Necrosis

Necrosis, the predominant type of non-regulated cell death, is immunogenic. Necrosis involves loss of plasma membrane integrity, resulting in the release of danger-associated molecular patterns (**DAMPs**) into the extracellular space. DAMPs activate the innate immune system to instigate further inflammation and can also act as chemotactic stimuli for inflammatory cells.

### Apoptosis

Apoptosis is regulated non-immunogenic cell death, in which plasma membrane integrity is retained throughout the process. Apoptosis has roles in development, homeostasis, and infection. The irreversible apoptotic route is mediated by executioner caspases, in particular caspase-3, and results in DNA fragmentation induced by caspase activation of DFF40,^45^ nuclear breakdown following caspase cleavage of nuclear envelope proteins,^67^ and cell shrinkage associated with cytoskeletal reorganisation.^55^ Histologically, apoptotic cells are pyknotic with hypereosinophilic cytoplasm and free or phagocytosed apoptotic bodies.^29^

Apoptosis can be instigated through intrinsic or extrinsic pathways. *Extrinsic apoptosis* begins at the plasma membrane, where external ligands, such as TNF, induce clustering of death receptors and processing of caspase-8. As an initiator caspase, caspase-8 can directly process and activate executioner caspases. *Intrinsic apoptosis* can be induced by various stressors and is driven from the mitochondria with the decompartmentalization of the inner mitochondrial contents, classically cytochrome c. Cytochrome c is able to bind directly to Apaf-1, inducing formation of the apoptosome and coordinating the “tilted-disk” caspase recruitment domain (**CARD**) complex.^19^ The initial helical structure formed by the Apaf-1 CARDs is extended by the binding of the caspase-9 CARD and the induced proximity cross-activates the caspase-9 catalytic activity. As with the extrinsic pathway, executioner caspase activation follows and then apoptotic cell death. Apoptosis is considered immunologically silent when the dying cell is phagocytosed, but can otherwise proceed to secondary necrosis if clearance does not happen promptly.^76^ Alternatively, in the presence of gasdermin E, executioner caspases can induce a switch from apoptotic to pyroptotic cell death.^70^

In human ARDS, soluble Fas ligand is released into airspaces and induces apoptotic cell death of pneumocytes via the extrinsic pathway, contributing to lung damage.^52^ In contrast to increased apoptosis of epithelial cells, alveolar neutrophils in human ARDS have a pro-survival phenotype with delay of apoptosis.^39^ Alveolar macrophages efferocytose apoptotic cells in health. However, under conditions such as ARDS (in which macrophages are converted to a pro-inflammatory phenotype and/or are overburdened by cell death), apoptotic cells are cleared less effectively, allowing secondary necrosis to take place.^50^

### Pyroptosis

Pyroptosis is gasdermin-mediated, regulated, lytic cell death and is highly immunogenic. In the context of infectious disease, this process can be initiated by oligomerization of various nucleotide-binding oligomerization domain-like receptors, including NLRP3 or AIM2-like receptors, to form inflammasome complexes. The signal is amplified by the recruitment and filamentation of multiple apoptosis-associated speck-like protein containing a CARD (ASC), which activates caspase-1 via CARD:CARD interactions and induced proximity.^47^ Non-canonical inflammasome formation is initiated by activating the caspase-4/-5/-11 group by cytoplasmic LPS. Inflammasome-activated caspases specifically process precursors of the cytokines IL1β and IL18, and also recruit gasdermin D, resulting in its cleavage into C- and N-terminal domains.^93^ The N-terminal segment of gasdermin D is post-translationally palmitoylated, leading to plasma membrane recruitment and then pore formation.^26^ Gasdermin D pores allow water to enter the cell across an osmotic gradient, leading to cell swelling and mechanical membrane rupture induced by the protein NINJ1.^21^ The caspase-cleaved interleukins can be released by the initial gasdermin D pores and then by membrane rupture. Several molecules targeting the NLRP3 inflammasome have been identified in experimental models for their therapeutic efficacy in reducing ALD.^46^ In a mouse inhalational-LPS-ALI model, apolipoprotein 3, a protein elevated in human COVID-19 patients with ALI, promoted macrophage pyroptosis through calcium-dependent ROS production and NLRP3 inflammasome activation.^63^

### Ferroptosis

Ferroptosis is lytic immunogenic cell death via iron-dependent lipid peroxidation by ROS. The lipid peroxidation can occur by enzymatic and non-enzymatic processes, meaning that changes in both enzyme availability and free-radical burden contribute to rates of ferroptosis.^103^ The lipid peroxidation dysregulates ion channels, causing cell swelling and activation of the protein NINJ1 and resulting in mechanical cell membrane rupture, analogous to the final stages of pyroptosis. The ferroptosis inhibitor, ferrostatin-1, resulted in a partial reduction in histologic lung injury score and detrimental ultrastructural cellular changes in a mouse model of sepsis-ALI.^15^

### Necroptosis

Necroptosis occurs following the activation by phosphorylation of mixed-lineage kinase domain-like pseudokinase (**MLKL**), which then forms an unregulated ion channel in the cell membrane that results in cell swelling as water moves along osmotic gradients. Cell rupture releases DAMPs into the surrounding tissue, which are recognized by PRRs on adjacent cells, inducing inflammation. The protein immediately upstream of MLKL in this pathway is RIPK3, which contains a receptor homology interacting motif (**RHIM**), and can be activated by the 3 other proteins known to contain a RHIM: TRIF, RIPK1, and ZBP1.^4,44,68^ TRIF is activated downstream of the Toll-like receptors TLR4 and TLR3. TLR4, a membrane-bound PRR whose canonical ligand is LPS, is also reported to respond to a variety of DAMPs, including high mobility group box-1. TLR3 is an endosomal membrane-bound PRR that responds to double-stranded viral RNA. Both TLRs induce other pro-inflammatory effects in addition to activating necroptosis. RIPK1 acts downstream of death receptor clustering and can induce necroptosis via RIPK3 under specific circumstances, such as the inhibition of caspase-8-mediated apoptosis. The complex regulation of RIPK1 places it as a situational effector of NF-κB activation, resulting in apoptosis or necroptosis, depending on the circumstances of the cell. Mice deficient in apoptotic executioner caspases 3 and 7, died in a necroptotic-dependent manner in a TNF-induced SIRS model.^27^ ZBP1 was initially regarded as a responder to viral Z-form DNA, but has since been implicated in responding to other non-self nucleic acid structures.

Necroptosis contributes to ALD in multiple ways. RIPK3 mediates aspects of lung damage in ventilator-induced lung injury, given that RIPK3 is elevated in the plasma of mechanically ventilated patients and RIPK3-deficient mice are protected from ventilator-induced lung injury.^75^ Elevated plasma RIPK3 levels in 5 prospective cohorts of human ICU patients also were associated with in-hospital mortality and organ failure.^48^ Alveolar epithelial death in response to LPS-induced lung injury in a mouse model was primarily via necroptosis rather than apoptosis.^82^ Necroptosis has been suggested as a major source of immune-mediated lesions in SIRS because RIPK3-deficient mice are protected from the systemic clinical effects of TNF-induced SIRS—as measured by survival, liver histology, and cytokine release.^27^ Mice deficient in pyroptosis-related caspase-1, or the apoptotic executioner caspases 3 and 7, experienced the same clinical effects compared with wild-type mice.^27^ In experimental rats, renal ischemia resulted in remote necroptosis within the lung.^102^

## Visualizing cell-death pathways with histopathology

Cell-death plasticity complicates the analysis of cell-death processes in diagnostic tissues involving multiple death pathways and tissue types. Morphologically, necrosis and immunogenic programmed cell death (pyroptosis, necroptosis, ferroptosis) appear identical in H&E-stained histologic sections. The practical application of visualizing programmed cell-death pathways in diagnostic pathology is limited, mainly because of the lack of pathway markers suitable for use in fixed tissue sections.^85^ Monoclonal antibodies to cleaved pMLKL, cleaved gasdermin D, and ferroptosis pathway components have provided pathway insights in mouse models,^51,90,104^ but have limitations in cross-species analysis. Molecular techniques involving spatial resolution on a histologic scale, such as spatial transcriptomics and multiplex fluorescence in situ hybridization, are also valuable tools for visualizing the spatial localization of inflammation and cell-death pathways during ALI, but are not readily available in diagnostic contexts.

## Implications for future vet ARDS research and translational medicine

### Extracellular vesicles in ARDS

Extracellular vesicles (**EVs**) are small membrane-bound vesicles secreted by cells into the extracellular space in both homeostatic and pathologic conditions. The vesicles contain cellular contents, such as proteins, mRNAs, miRNAs, DNA, and lipids, which can impact the recipient cell.^42^ Liposomes are artificially created EVs for therapeutic purposes. Lung-targeted liposomes are being developed as a drug-delivery method to enable precision application of therapeutics in human ARDS.^49^ At this stage, the therapeutic impact of EVs in vetARDS is limited to pre-clinical experimental models. Detailed knowledge of the ARDS endotypes and their underpinning pathogeneses supports identification of future therapeutic targets. For example, EVs derived from LPS-stimulated macrophages induced pyroptosis by NLRP3 activation in airway epithelial cells in mouse lungs,^36^ whereas mesenchymal stem-cell-derived EVs downregulated pyroptosis, reducing lung injury in a mouse model of cardiopulmonary bypass-induced experimental ALI.^101^

## The comparative innate immunology of ARDS

**Figure 4.**
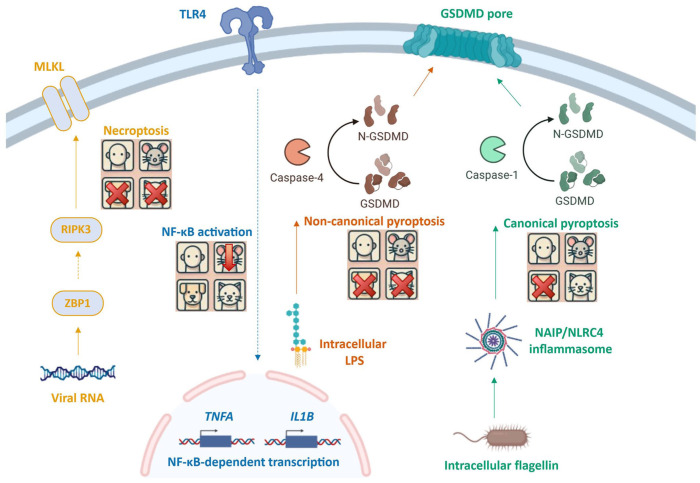
Innate immune responses vary between species. **Necroptosis** is initiated following the recognition of non-self RNA by Z-DNA binding protein 1(ZBP1). *Carnivora* lack the pore-forming protein mixed lineage kinase domain-like pseudokinase (MLKL) and cannot complete this pathway. **LPS signaling:** Toll-like receptor 4 (TLR4) is activated by lipopolysaccharide (LPS), in all species. The sensitivity of cells to LPS is species-dependent, and a pronounced decrease in sensitivity is observed in mice. **Non-canonical pyroptosis:** Intracellular LPS activates the caspase-4 family, leading to cleavage and activation of gasdermin D (GSDMD). The caspase-1/-4 hybrid gene found in cats and dogs has lost this functionality. **Canonical pyroptosis:** Intracellular bacteria expressing flagella can be recognized by the NLR family CARD domain-containing protein 4 (NLRC4)/NLR family apoptosis inhibitory protein (NAIP) inflammasome, followed by caspase-1 activation. The *Naip/Nlrc4* genes are simultaneously lost in many species, including the dog. In these cases, gasdermin D-dependent cell death will not occur in response to intracellular flagellin. RIPK3 = receptor-interacting serine/threonine-protein kinase 3.

The development of precision therapeutics based on the ARDS endotypes will involve targeting specific innate immune pathways. Different evolutionary pressures have led to species-specific differences in expression of innate immune components (**Fig. 4**). Recognition of these differences is important to accurately interpret data or translate therapeutics between humans and veterinary species. The use of inhibitors targeting the necroptosis mediators RIPK3 and MLKL is of interest in human ARDS therapeutics.^35^ However, *Carnivora*, including dogs and cats, lack a functional MLKL,^24^ making this target unfeasible. Likewise, the protective effects of RIPK3 inhibition, observed in mice, may not be translatable to other species.

LPS can induce systemic inflammatory response ARDS, but mice have a pronounced decrease in sensitivity to LPS compared with humans,^18,53^ partially because of protein structure differences in the TLR4 co-receptor MD-2.^88,89^ Dogs and cats have lost the ability to respond to cytoplasmic LPS via caspase-4 because they have a non-functional hybrid caspase-1 and -4 fusion protein.^23^ NLRC4, which together with NAIP, a co-receptor, detects flagellin and related bacterial components, and has been implicated in ARDS lesions caused by pancreatitis and ischemia-reperfusion injury.^30^ However, the NLRC4/NAIP inflammasome has been lost in the dog, pig, and chicken.^8,28,72^ It remains undetermined how the loss of cytosolic LPS signaling in dogs and cats, and the loss of all NLRC4 inflammasome responses in the dog, impacts the development of ARDS.

The differences between inflammatory and cell-death pathways in humans, mice, dogs, and cats create intriguing research questions regarding species heterogeneity and ARDS progression. Leveraging these differences experimentally could help inform the discovery of new therapeutic targets to benefit both human and veterinary patients.

## Conclusion

ARDS endotypes are conceptually applicable to veterinary species, and work is required to now characterize the specific molecular signatures of the endotypes in veterinary contexts. In our pair of reviews, we highlight the complexity of the cellular processes involved in the primary injury and amplification to ARDS, and the interconnectedness of those pathways, which are further confounded by species-specific mechanistic differences. To achieve the aim of using ARDS endotypes to create precision medicine, we should aspire to precision in the measurement of experimental and observational evidence in models that retain interconnected pathways. For example, measuring multiple cell-death pathways concurrently within complex models, using data or material from clinical cases, may facilitate our appreciation of the cross talk between processes on a cellular and tissue level. This interconnectedness also underscores the importance and value of a multidisciplinary approach—both in experimental design and in the analysis of individual clinical cases.
